# Weight loss interventions for Hispanic women in the USA: a protocol for a systematic review

**DOI:** 10.1186/s13643-019-1213-3

**Published:** 2019-12-01

**Authors:** Kristin E. Morrill, Melissa Lopez-Pentecost, Guadalupe Ballesteros, Jeanne L. Pfander, Melanie D. Hingle, Yann C. Klimentidis, Cynthia A. Thomson, David O. Garcia

**Affiliations:** 1 Department of Nutritional Sciences, College of Agriculture & Life Sciences, University of Arizona, Tucson, AZ USA; 2 Department of Clinical and Translational Sciences, College of Medicine, University of Arizona, Tucson, AZ USA; 3 Department of Physiology, College of Medicine, University of Arizona, Tucson, AZ USA; 4 University Libraries, University of Arizona, Tucson, AZ USA; 5 Department of Epidemiology and Biostatistics, University of Arizona, Mel and Enid Zuckerman College of Public Health, Tucson, AZ USA; 6 Department of Health Promotion Sciences, University of Arizona, Mel and Enid Zuckerman College of Public Health, Tucson, AZ USA

**Keywords:** Hispanic, Weight loss, Culturally sensitive, Intervention, Diet, Physical activity

## Abstract

**Background:**

In the U.S., Hispanic women experience a disproportionate rate of obesity and obesity-related chronic diseases. At the same time, Hispanic women remain considerably underrepresented in behavioral weight loss interventions. The purpose of this review is to systematically evaluate the evidence related to the effectiveness of weight loss interventions among Hispanic women in the U.S. This review will identify elements of successful weight loss interventions as well as areas for future research.

**Methods/Design:**

The following databases will be searched to identify all relevant articles (from inception onwards): PubMed, Embase, Scopus, Web of Science (Science Citation Index and Social Sciences Citation Index), PsycINFO, CINAHL, Chicano Database, SPORTDiscus, CAB Abstracts, and Google Scholar. We will include randomized controlled trials and quasi-experimental studies of adult women (> 18 years) from Hispanic/Latino background living in the United States. Eligible interventions will target weight-related behaviors (including diet, physical activity, behavior modification and/or their combinations). The review’s primary outcome will be weight change (expressed as change in lbs/kg or body mass index (BMI) (kg/m^2^)). Three reviewers will independently screen and select data and two will extract data. The methodological quality (or risk of bias) of individual studies will be appraised using the Effective Public Health Practice Project Quality Assessment Tool. A narrative synthesis will describe quality and content of the evidence.

**Discussion:**

The aim of this systematic review is to critically examine existing weight loss interventions for Hispanic women in the U.S. and provide quality evidence for the effectiveness of these interventions on weight loss. Further, this review seeks to identify characteristics of effective interventions and suggest future directions for research efforts targeting weight loss in this population. This review will inform the development of future weight loss interventions for this population.

**Systematic review registration:**

PROSPERO CRD42019119094

## Background

Hispanic women will comprise 25% of all women in the USA by 2050 if current population growth trends continue [1, 2]. Hispanic women experience disproportionate rates of obesity with a rate of 50% compared with 38% in non-Hispanic White (NHW) women [3]. Obesity is associated with many chronic diseases and health conditions including hypertension, type 2 diabetes, chronic liver disease, heart disease, several cancers, body pain, depression, and overall low quality of life [4–8]. Consequently, Hispanic women are more likely to develop type 2 diabetes and chronic liver disease compared with NHW women and are significantly more likely to die from these conditions [9, 10]. Efforts to improve modifiable lifestyle behaviors, such as diet and physical activity, to reduce the obesity burden and improve health in Hispanic women, are urgently needed.

Current guidelines for weight management include the use of comprehensive lifestyle interventions consisting of diet, physical activity, and behavior therapy [11]. When effective, these interventions typically result in a weight loss of approximately 18 lbs. in a 6-month period or 1–2 lbs. per week [11]. However, long-term maintenance of weight loss remains a challenge as short-term weight loss is typically followed by a regain of 30–50% of initial body weight over the subsequent 2–3 years [12, 13]. Within the field of behavioral weight loss interventions, Hispanic women remain considerably underrepresented. A recent systematic review found that Hispanics in general have comprised less than 10% of participants in behavioral weight loss interventions [14]. One of the largest lifestyle interventions to include Hispanic women, the Diabetes Prevention Program (DPP), was a landmark trial that demonstrated a comprehensive weight loss intervention can reduce incidence and progression of diabetes [15]. By the completion of the study, weight loss in the lifestyle arm was similarly effective in Hispanic women and NHW women with − 5.9% and − 4.5% loss of initial body weight after 30 weeks, respectively [16]. The success of the lifestyle arm in inducing short-term weight loss in Hispanic women was likely due, in part, to the range of culturally sensitive materials and strategies implemented to take into consideration the ethnic diversity of study participants (e.g., the use of community lay workers often culturally matched to participants, culturally sensitive cooking materials) [16–18]. Although DPP was a large trial, only 16% (*n* = 154) of the intensive lifestyle intervention (ILI) group identified as Hispanic women and information including level of acculturation, years in the USA, and country of origin were not collected, thereby limiting generalizability to different sub-populations within the Hispanic community [16]. In the Look AHEAD trial, the longest randomized trial to evaluate the effectiveness of lifestyle modification on weight and cardiovascular-related health outcomes, Hispanic women comprised only 9% (*n* = 240) of participants in the ILI group and weight loss achieved by year 1 was slowly regained over the next 3 to 7 years, including among Hispanic women [19, 20]. Additionally, similar to the DPP, the multicomponent lifestyle intervention delivered to participants makes it difficult to ascertain which specific intervention components were effective in inducing the observed weight loss.

Our hypothesis is that Hispanic women are uniquely and importantly different from NHW women when establishing effective programs for weight management. Cultural, social, and economic factors shape the way Hispanic women think and act regarding diet, physical activity, and weight loss. Further, actual and perceived barriers Hispanic women face while attempting to implement lifestyle changes need to be considered as influential elements in developing effective interventions. For example, among Mexican-American women, weight loss efforts may be influenced by a desire for a curvier figure, a desire to feel a part of American society, social hierarchies found within the home, concerns over spending time on themselves, familial pressures, and a lack of social/familial support [21, 22]. Additionally, structural barriers to diet and physical activity, such as the built environment, food deserts, and related issues in regards to access to healthy food may impede efforts to lose weight [23–25]. The process of acculturation has been identified as a factor affecting diet and physical activity behaviors both positively and negatively in individuals immigrating to the USA [26]. For Hispanic women, greater acculturation is positively associated with greater levels of total physical activity throughout the day [27–29] and increased likelihood of engaging in recommended amount of physical activity [30]. While data are limited, westernized dietary acculturation for Hispanic women is characterized as increased intakes of saturated fat, sugar, dessert, and low-fat milk, and decreased intake of corn tortillas, low-fiber bread, and high-fat milk [31]. Given this evidence, the role of socio-cultural factors such as level of acculturation and immigrant status remains important to understand and assess while seeking to improve weight loss efforts in Hispanic women. Additionally, woven into these factors are specific values, customs, and perceptions rooted in cultural gender norms that affect the engagement of Hispanic women in weight loss efforts [21, 25]. For example, Hispanic women may perceive a fuller figure to be more “healthy-looking” and desirable and may be deterred from engaging in physical activity by their husbands [21, 25, 32, 33]. Hispanic women may find it difficult to adopt healthier eating and cooking habits that would promote weight control for fear of the impact these changes would have on their family [25]. These unique barriers faced by Hispanic women attempting to engage in healthy lifestyle behaviors warrant interventions that are tailored to the needs of Hispanic individuals in general but more specifically, the needs of Hispanic women. For this reason, we have opted to focus this review on weight loss interventions that include only Hispanic women.

To date, systematic reviews focused exclusively on U.S. Hispanic women exist for diabetes risk factors management [34], cancer screening [35, 36], factors associated with physical activity [37], and maternal health and breastfeeding [38]. Multiple reviews have examined only physical activity interventions in Hispanic adults [39–41]. In a systematic review by Corona et al., authors examined lifestyle interventions in adult Latinas but did not summarize information related to the effectiveness of the interventions in inducing weight loss, study socio-economic factors, and recruitment variables, and no quality assessment of studies was performed [42]. The current review will extend the literature by providing a comprehensive and rigorous examination of weight loss interventions in Hispanic women and will include an in-depth synthesis of participant characteristics, intervention design, and study quality while using a predetermined measure for intervention success.

The purpose of this systematic review is to characterize previously tested weight loss interventions in Hispanic women in the USA and to identify areas for future research. Additionally, components of successful interventions (those that have achieved clinically meaningful weight loss of ≥ 3% [11]) will be identified so that future interventions can build on previous findings and ensure meaningful progress is made. A weight loss of ≥ 3% is associated with clinically meaningful reductions in risk factors for diabetes such as hemoglobin A1c and blood glucose [11] and therefore was chosen as a predetermined measures of intervention effectiveness.

In the current manuscript, the term “Hispanic” is representative of individuals who classify themselves as a person of Mexican, Cuban, South or Central American, Puerto Rican, or other Spanish culture or origin, regardless of race. Importantly, the ongoing Hispanic Community Health Study/Study of Latinos (HCHS/SOL) continues to provide new insights into factors involved in the prevention and treatment of chronic disease among Hispanic/Latino persons from different countries of origin [43, 44]. We acknowledge the considerable heterogeneity of the term “Hispanic” and will recognize other terms (e.g., Latino/a/x) and/or subgroups (e.g., Mexican American) within our search strategy in efforts to be as inclusive as possible.

## Methods and analysis

### Study registration

This systematic review protocol is being reported in accordance with the reporting guidance in the Preferred Reporting Items for Systematic Reviews and Meta-Analyses Protocol (PRISMA-P) statement [45]. The PRISMA checklist is provided in Additional file 1. As a systematic review of published studies, ethics approval will not be required. This review has been registered with the International Prospective Register of Systematic Reviews (PROSPERO) (registration number: CRD42019119094; date of registration: April 2, 2019.

### Study eligibility criteria

Studies will be selected according to the criteria outlined in Table 1.

**Table 1:** Inclusion and exclusion criteria

PICOS strategy	Inclusion criteria	Exclusion criteria
P - Population	Hispanic women, 18+ years old, living in the USA	Studies that recruited both men and women (however, studies that included men and children as a strategy to engage women will be kept), studies focused on children and/or adolescents that allowed parents to attend, patients who are hospitalized or institutionalized, patients with eating disorders, patients who have recently undergone bariatric surgery
I - Intervention	Lifestyle interventions ≥ 12 weeks in duration, targeting diet, and/or physical activity to reduce body weight	Surgical procedures, nonsurgical devices and procedures, pharmacological treatments, complementary/alternative treatments, dietary supplements intended for weight loss, population-focused health promotion campaigns, and interventions that do not focus on modifying weight, interventions to prevent excessive weight gain during pregnancy
C - Comparison	For RCTs, wait-list control or usual care For quasi-experimental, no comparison required	
O - Outcome	Studies reporting objectively measured weight change (expressed as change in lbs. or kg or BMI (kg/m^2^)) as a primary or secondary outcome.	Self-reported measures of weight change
S - Study design	RCTs and quasi-experimental studies	Reviews, observational studies (cross sectional, case-control, and cohort studies), case reports, case series, in vitro studies, animal studies, secondary analyses of trials, and survey development studies

#### Study participants

Our decision to focus our review on Hispanic women living in the USA stems from the knowledge that U.S. Hispanic women have unique attitudes, barriers, and facilitators related to weight loss with important socio-cultural contextual factors specific to the USA (e.g., acculturation, built environment, high burden of obesity, and related disease) meriting focused examination. Additionally, racial/ethnic- and sex-specific models exploring predictors of weight loss have demonstrated differences in key predictors of weight loss, further justifying the need to independently focus on Hispanic women [20].

#### Interventions

Lifestyle interventions ≥ 12 weeks in duration, a minimum timeframe associated with short-term weight loss [46], targeting diet and/or physical activity to reduce body weight will be included. This will include dietary interventions targeting weight, physical activity interventions targeting weight, and a combination of both with and without behavioral modification. Dietary interventions will be defined as interventions where diet is modified through changes in diet-related behaviors (e.g., portion control, stimulus control, cooking skills). Physical activity interventions will be defined as interventions where physical activity is modified through changes in physical activity-related behaviors (e.g., increasing leisure time physical activity, reducing sedentary behavior). Interventions will not be excluded based on intervention delivery modality, but modality will be tracked as a component that may influence program effects. Additionally, interventions delivered by a range of different interventionists (e.g., students, physicians, community health workers) will be included.

### Outcomes and prioritization

Studies to be included in this review must report weight or body mass index (BMI) (kg/m^2^) as a primary or secondary outcome. Changes in risk factors for chronic disease (e.g., blood glucose, hemoglobin A1c, and lipids) included as primary or secondary outcomes in eligible studies will be summarized. Characteristics of interventions that have induced a clinically meaningful weight loss of ≥ 3% [11] will be summarized.

### Data sources and search strategy

The following databases will be searched by a professional librarian (JLP) for all relevant articles from inception onwards: PubMed, Embase, Scopus, Web of Science (Science Citation Index and Social Sciences Citation Index), PsycINFO, CINAHL, Chicano Database, SPORTDiscus, CAB Abstracts, and Google Scholar. A detailed description of the search strategy for PubMed is included as Additional file 2. Reference lists from all eligible studies will be hand-searched in addition to reference lists of related reviews. The search will be supplemented with suggestions from experts in the field. The search will be limited to publications written in the English language.

### Data management

Results from the search through the electronic databases will be uploaded by JLP into EndNote citation manager software and duplicates removed. The EndNote file will then be uploaded to Covidence systematic review software (Veritas Health Innovation, Melbourne, Australia). KM, MLP, and GB will then independently begin the reviewing process.

### Selection process

Excluding duplicates, articles generated from the search strategy will be divided into three equal sections (A, B, C). Then, three authors (KM, MLP, GB) will independently perform an initial title and abstract review in the following manner: KM will review A+B, MLP will review B+C, and GB will review A+C. The three authors will then meet and results will be discussed until a consensus is reached regarding eligible studies. In the next step, remaining articles will be read in full-text format independently by KM and MLP. Discrepancies at these two screening steps will be resolved by DG and the set of articles to be reviewed will be finalized.

### Data extraction

Data from these articles will be extracted independently by KM and MLP and reviewed by GB. Extracted data from the articles will be collected by KM and MLP using a standardized template form developed specifically for this review and cross-referenced by GB for any discrepancies. If data extraction for an eligible study cannot be accomplished due to information inadequately described or missing in the full-text article, KM will contact the publication’s corresponding author via email up to three times to request the information. KM and MLP will pilot the standardized template on a subsample of eligible studies and make any appropriate adjustments to the data collection fields as necessary before continuing with the remaining studies. In the case there are multiple publications for an eligible study, data will be extracted from each manuscript in order to retrieve all information relevant to this review. Intervention materials, such as educational materials provided to participants, will be requested from trial authors and published as supplementary materials in the final review [47].

Data to be extracted from eligible articles are as follows:

1. Author and year of publication
2. Participant population (age, important eligibility criteria such as weight classification or disease state)
3. Socio-cultural factors (acculturation level and Hispanic subgroup or country of origin, if available)
4. Study design (randomized controlled trial or quasi-experimental design)
5. Recruitment variables (setting, strategy, and effectiveness)
6. Intervention characteristics (duration, study materials, study delivery setting, intervention modality, theoretical frameworks/behavior change techniques, and culturally sensitive strategies implemented)
7. Comparator (if available) and description
8. Study outcome information (weight loss and/or changes in BMI included as primary or secondary outcome) and changes in other risk factors for chronic disease (e.g., blood glucose, hemoglobin A1c, and lipids) included as the secondary study outcomes (if available)
9. Retention and adherence rates

### Quality assessment

Quality in all studies will be independently assessed by KM and MLP using the Effective Public Health Practice Project Quality Assessment Tool (EPHPP) [48]. This standardized tool, developed by the Effective Public Health Practice Project in Canada, was chosen since it can be used to evaluate a range of intervention study designs including RCTs and pre- and post-studies, both of which may be included in the review. The EPHPP evaluates study quality by assessing the following six domains: (1) selection bias; (2) study design; (3) confounders; (4) blinding; (5) data collection; and (6) withdrawals/dropouts. When using this tool, each of these six domains is rated from weak (1 point) to strong (3 points) and these are averaged to provide a total quality score for the study. Validity and reliability for this tool meets accepted standards [48]. For the current review, all studies to be included in the review will have a copy of the EPHPP attached, and both KM and MLP will independently assess each article. Any discrepancies will be resolved by DG. Because we are not conducting a meta-analysis, there will be no data synthesis, and therefore, no sensitivity analysis will be conducted with the results from this assessment.

### Data analysis

A narrative synthesis of eligible studies will be conducted. This will include qualitatively summarizing all extracted data. Quantitative summaries of extracted data such as the proportion of studies to achieve a clinically meaningful weight loss of ≥ 3% [11] and frequencies of specific intervention characteristics (e.g., use of theoretical framework, study delivery setting, culturally sensitive strategies implemented) will be reported. KM will synthesize all of the data and this will be reviewed by MLP. Discrepancies will be resolved by DG. These summaries will be used to assess the effectiveness of weight loss interventions for Hispanic women in the USA and address gaps in the existing literature. After synthesizing the data, we will identify areas for future research.

## Discussion

This review will be among the first to provide quality evidence for the effectiveness of weight loss interventions developed specifically for Hispanic women in the USA and identify salient features of effective interventions. This information will be useful to inform the development of future weight loss interventions for this population. Potential limitations include a limited number of eligible studies, studies with small samples sizes, insufficient detail of sample eligibility, and/or intervention strategies and characteristics as well as the exclusion of studies published in non-English languages. Any amendments made to this protocol while conducting the study will be described in PROSPERO and outlined in the final published manuscript. Findings from this review will be widely disseminated through conference presentations and peer-reviewed publications.

## Supplementary information

**Additional file 1.** PRISMA-P (Preferred Reporting Items for Systematic review and Meta-Analysis Protocols) 2015 checklist: recommended items to address in a systematic review protocol.

**Additional file 2.** Morrill SR PubMed Search Strategy (07/02/2019).

## Data Availability

Data sharing is not applicable to this article as no datasets were generated or analyzed during the current study.

## Abbreviations

BMI: Body mass index
CINAHL: The Cumulative Index to Nursing and Allied Health Literature
DPP: Diabetes Prevention Program
EPHPP: Effective Public Health Practice Project Quality Assessment Tool
PRISMA-P: Preferred Reporting Items for Systematic Reviews and Meta-Analyses Protocol
PROSPERO: International Prospective Register of Systematic Reviews
RCTs: Randomized controlled trials
SCI: Science Citation Index
SSCI: Social Sciences Citation Index
