# Supplementary material for: Weight loss interventions for Hispanic women in the USA: a protocol for a systematic review
Source: Syst Rev. 2019 Dec 1;8:301. doi: 10.1186/s13643-019-1213-3 (PMC6886178; doi:10.1186/s13643-019-1213-3)
Supplement: Supplementary file 2 — Additional file 2. Morrill SR PubMed Search Strategy (07/02/2019). [file 13643_2019_1213_MOESM2_ESM.docx]

**Morrill SR PubMed Search Strategy (07/02/2019)**

**HISPANIC**

"Hispanic Americans"[Mesh]

OR Hispanic[Title/Abstract] OR Hispanics[Title/Abstract] OR Central American[Title/Abstract] OR Central Americans[Title/Abstract] OR Chicano[Title/Abstract] OR Chicanos[Title/Abstract] OR Chicana[Title/Abstract] OR Chicanas[Title/Abstract] OR Cuban[Title/Abstract] OR Cubans[Title/Abstract] OR Dominican[Title/Abstract] OR Dominicans[Title/Abstract] OR Latin American[Title/Abstract] OR Latin Americans[Title/Abstract] OR Latin[Title/Abstract] OR Latina[Title/Abstract] OR Latinas[Title/Abstract] OR Latino[Title/Abstract] OR Latinos[Title/Abstract] OR LatinX[Title/Abstract] OR Mexican[Title/Abstract] OR Mexicans[Title/Abstract] OR Puerto Rican[Title/Abstract] OR Puerto Ricans[Title/Abstract] OR South American[Title/Abstract] OR South Americans[Title/Abstract] OR Spanish speaker[Title/Abstract] OR Spanish speakers[Title/Abstract] OR Spanish speaking[Title/Abstract]

**WOMEN**

"Women"[Mesh] OR "Female"[Mesh]) OR "Mothers"[Mesh]

OR woman[Title/Abstract] OR women[Title/Abstract] OR female[Title/Abstract] OR females[Title/Abstract] OR feminine[Title/Abstract] OR mother[Title/Abstract] OR mothers[Title/Abstract] OR daughter[Title/Abstract] OR daughters[Title/Abstract] OR grandmother[Title/Abstract] OR grandmothers[Title/Abstract] OR aunt[Title/Abstract] OR aunts[Title/Abstract] OR sister[Title/Abstract] OR sisters[Title/Abstract] OR mujer[Title/Abstract] OR mujeres[Title/Abstract]) OR madre[Title/Abstract] OR madres[Title/Abstract] OR abuela[Title/Abstract] OR abuelas[Title/Abstract] OR tia[Title/Abstract] OR tias[Title/Abstract] OR hermana[Title/Abstract] OR hermanas [Title/Abstract]

**WEIGHT**

"Body Weight Changes"[Mesh] OR "Body Mass Index"[Mesh] OR "Body Weight"[Mesh:NoExp] OR "Body Weight Maintenance"[Mesh] OR "Ideal Body Weight"[Mesh] OR "Overweight"[Mesh] OR "Overnutrition"[Mesh] OR "Waist Circumference"[Mesh] OR "Waist-Height Ratio"[Mesh]

OR body mass index[Title/Abstract] OR BMI[Title/Abstract] OR body weight[Title/Abstract] OR overweight[Title/Abstract] OR over nutrition[Title/Abstract] OR overnutrition[Title/Abstract] OR weight gain[Title/Abstract] OR weight loss[Title/Abstract] OR weight maintenance[Title/Abstract] OR weight management[Title/Abstract] OR obesity[Title/Abstract] OR obese[Title/Abstract] OR waist circumference[Title/Abstract] OR waist-height ratio[Title/Abstract] OR waist-to-height ratio[Title/Abstract]

**INTERVENTIONS**

"Diet, Reducing"[Mesh] OR "Weight Reduction Programs"[Mesh] OR "Diet"[Mesh]

OR reducing diet[Title/Abstract] OR reducing diets[Title/Abstract] OR diet[Title/Abstract] OR diets[Title/Abstract] OR dietary[Title/Abstract] OR weight reduction[Title/Abstract] OR weight loss[Title/Abstract] OR losing weight[Title/Abstract] OR eating habit[Title/Abstract] OR eating habits[Title/Abstract] OR food intake[Title/Abstract] OR food restriction*[Title/Abstract] OR eating behavior[Title/Abstract] OR eating behaviors[Title/Abstract]

OR “Physical Exertion"[Mesh] OR "Physical Fitness"[Mesh] OR "Exercise Therapy"[Mesh] OR "Exercise"[Mesh] OR "Sports"[Mesh] OR "Physical Education and Training"[Mesh]

OR (movement*[Title/Abstract] OR physical exertion[Title/Abstract] OR physical activity[Title/Abstract] OR physical activities[Title/Abstract] OR physical fitness[Title/Abstract] OR sport[Title/Abstract] OR sports[Title/Abstract] OR exercis*[Title/Abstract]

OR "Women's Health Services"[Mesh] OR "Health Promotion"[Mesh] OR "Life Style"[Mesh]

OR intervention*[Title/Abstract] OR health promotion*[Title/Abstract] OR life style*[Title/Abstract] OR lifestyle*[Title/Abstract]

**AGE**

NOT ("Child"[Mesh] OR "Infant"[Mesh] OR "Adolescent"[Mesh]) NOT "Adult"[Mesh]

**FINAL SEARCH (combining above listed search sets):**

HISPANIC AND WOMEN AND WEIGHT AND INTERVENTIONS AND AGE

**Limit: English language only**
